# Combining multimodal medical imaging and artificial intelligence for the early diagnosis of pancreatic cancer

**DOI:** 10.3389/fmed.2025.1631671

**Published:** 2025-08-08

**Authors:** Xiaoli Yu, Yuyin Wang, Yunchun Wang, Junbang Feng, Shimei Fan, Chuanming Li

**Affiliations:** ^1^Medical Imaging Department, Chongqing Emergency Medical Center, Chongqing University Central Hospital, School of Medicine, Chongqing University, Chongqing, China; ^2^Medical Imaging Department, Beijing Anzhen Nanchong Hospital of Capital Medical University & Nanchong Central Hospital, Sichuan, Chongqing, China; ^3^Physical Examination Center, Chongqing Emergency Medical Center, Chongqing University Central Hospital, School of Medicine, Chongqing University, Chongqing, China

**Keywords:** pancreatic cancer, artificial intelligence, multimodal imaging, early diagnosis, DICOM

## 1 Introduction

Pancreatic cancer is a prevalent digestive system malignancy that poses a significant threat to human health. It ranks among the most lethal cancers, with an overall 5-year relative survival rate of ~11% (1). This disease is distinguished by non-specific early symptoms, high invasiveness, high mortality, and low curability. In 2024, pancreatic cancer is projected to cause ~66,440 new cases and ~51,750 deaths, making it the third leading cause of cancer-related death, surpassing breast cancer (2). According to global cancer statistics, pancreatic cancer is projected to become the second leading cause of cancer-related death by 2030 (3, 4). The primary factor contributing to its high mortality rate is the lack of prominent early clinical symptoms, as well as the absence of definitive early diagnostic markers and effective screening strategies. Consequently, by the time of diagnosis, many patients have already progressed to advanced stages, missing the optimal treatment window (5, 6). However, if pancreatic cancer could be accurately diagnosed in its early stages, both survival and cure rates could be substantially increased (7). Thus, the development of objective methods for the early, rapid, and precise diagnosis of pancreatic cancer remains a critical challenge.

Currently, puncture biopsy remains the gold standard for diagnosing pancreatic cancer. However, it is invasive, costly, time-consuming, and associated with a high risk of complications such as infection, bleeding, and pancreatitis. Moreover, the heterogeneity of tumor tissues may compromise the representativeness of sampling, thereby limiting the accuracy of the test results. In recent years, the potential of non-invasive and comprehensive imaging techniques for the early diagnosis of pancreatic cancer has garnered significant research attention. Studies have demonstrated that multimodal medical imaging technologies, including endoscopic ultrasound (EUS), CT, and MRI, are widely applied during the preoperative evaluation of pancreatic cancer patients and have achieved promising results in early detection. Nevertheless, relying solely on imaging features for pancreatic cancer diagnosis heavily depends on physicians' visual assessments and diagnostic experience. This approach has several limitations, such as low sensitivity, atypical imaging characteristics of some pancreatic cancers, and susceptibility to misdiagnosis or missed diagnosis due to interobserver variability (8). With advancements in artificial intelligence (AI), AI-based models leveraging medical imaging technology have shown remarkable diagnostic accuracy and efficiency in assisting with pancreatic cancer diagnosis. By employing advanced algorithms to analyze large-scale medical image datasets, these models can rapidly and precisely identify subtle imaging features of pancreatic cancer that are imperceptible to the human eye, providing clinicians with reliable diagnostic references and significantly increasing the efficiency and quality of early pancreatic cancer diagnosis.

In this work, we argue that, considering the critical importance of early pancreatic cancer diagnosis and the limitations of existing diagnostic methods, organically integrating multimodal medical imaging technologies—such as EUS, CT, and MRI—with distinct imaging principles to leverage their complementary strengths and comprehensively extract pancreatic cancer-related information represents a key breakthrough for achieving early and rapid diagnosis. Building on this foundation, developing an innovative AI-assisted framework for early pancreatic cancer diagnosis holds substantial importance. This framework should not only enable deep mining of potential key features embedded in multimodal image data but also increase diagnostic accuracy and stability through algorithm optimization. The ultimate aim is to provide clinicians with fast and reliable decision support, enabling them to make more efficient and accurate judgments in complex pancreatic cancer diagnostic scenarios while offering a promising direction for future advancements in the precise diagnosis of early-stage pancreatic cancer.

## 2 Current status of artificial intelligence and multimodal medical imaging for the assisted diagnosis of pancreatic cancer

Early detection of pancreatic cancer remains an extremely challenging task. Owing to its high-resolution imaging ability, precise biopsy capability, and staging evaluation value, EUS has emerged as a critical tool for the early diagnosis, pathologic confirmation, and preoperative assessment of pancreatic cancer, particularly for small lesions, complex cases, or scenarios requiring minimally invasive interventions. Studies have demonstrated that EUS outperforms CT or MRI in diagnosing pancreatic cancer, with superior sensitivity for lesions ≤ 2 cm in diameter. Specifically, EUS can detect tumors as small as 5 mm in diameter, highlighting its significant advantages in this context (9, 10). Recent research has indicated that AI can enhance the performance of EUS, with AI-assisted EUS models achieving diagnostic accuracy surpassing or matching that of human interpretation (11). A retrospective study (12) analyzed EUS images from 216 patients using a support vector machine (SVM) model to differentiate normal tissue from pancreatic cancer. The results revealed an accuracy of 98%, sensitivity of 94.3%, and specificity of 99.5%. Furthermore, a systematic review of 11 studies evaluating AI-assisted EUS modeling for pancreatic cancer diagnosis reported overall accuracies, sensitivities, and specificities ranging from 80–97.5%, 83–100%, and 50–99%, respectively (13). On the basis of current evidence, AI-assisted EUS models demonstrate promising potential for early pancreatic cancer detection, characterized by high diagnostic accuracy, despite being in the early stages of development and clinical application. For example, the Aichi Cancer Research Center in Japan developed a deep learning-based AI model capable of distinguishing pancreatic cancer lesions from non-cancerous lesions using EUS images. In the validation set, this model achieved an area under the curve (AUC) of 0.90, a sensitivity of 0.94, and a specificity of 0.82 (14).

CT, which has superior spatial and temporal resolution, is widely recognized as the preferred non-invasive imaging modality for pancreatic cancer detection. It plays a critical role in diagnosing, staging, and evaluating treatment efficacy for patients with pancreatic cancer. Cao et al. introduced a deep learning framework named artificial intelligence for pancreatic cancer detection (PANDA), which was designed to detect and classify pancreatic lesions using non-contrast CT images. The model was trained on a single-center dataset of 3,208 patients' non-contrast CT scans. In a multicenter validation involving 6,239 patients from 10 centers, the model demonstrated exceptional performance, achieving an AUC ranging from 0.986 to 0.996 for lesion detection. This model is particularly advantageous for patients contraindicated for intravenous contrast (15). Mukherjee et al. developed a radiomic-based machine learning (ML) model for the prediagnostic detection of pancreatic cancer. Their study included prediagnostic CT scans from 155 patients with pancreatic cancer and 265 age-matched controls with a normal pancreas, and 34 imaging histologic features were selected. Validation was conducted on 176 in-house patients and 80 external controls. The SVM-based classifier achieved a sensitivity of 95.5% and an AUC of 0.98. These findings indicate promising results for early-stage detection, potentially improving outcomes by identifying tumors at a resectable stage (16). A study from Zhejiang University utilized abdominal-enhanced CT images from 319 patients to train a deep learning model capable of suggesting pancreatic tumor diagnoses on the basis of original abdominal CT images. The model achieved an AUC of 0.871 and an F1 score of 88.5%. Across all tumor types, the average diagnostic accuracy was 82.7%, with differential diagnostic accuracies of 100% for intraductal papillary mucinous neoplasms (IPMNs) and 87.6% for pancreatic ductal adenocarcinoma (PDAC) (17). Ma et al. screened 222 pathologically confirmed pancreatic cancer cases and 190 normal pancreas cases and trained a convolutional neural network (CNN) model for binary classification (presence or absence of pancreatic cancer) using 7,245 CT images. The model exhibited an accuracy of 95.47%, sensitivity of 91.58%, and specificity of 98.27%, showing no significant difference compared with radiologists (18). In summary, artificial intelligence-assisted CT imaging for pancreatic cancer diagnosis has garnered substantial attention in recent years, demonstrating promising potential for increasing diagnostic accuracy and improving clinical decision-making.

MRI is a widely used non-ionizing radiation examination technique in clinical practice and is characterized by high soft tissue contrast and spatial resolution. It can more accurately reflect changes in tumor tissue components, such as the degree of fibrosis, microvessel density, hypoxia, and other alterations in tissue status and composition. Multiparameter quantitative analysis of tumor tissues has gradually become an essential auxiliary tool for the early diagnosis of pancreatic cancer (19). Combined multisquence MRI (anatomical and functional) is widely recognized as a critical tool for diagnosing, staging, and evaluating treatment efficacy in pancreatic cancer, demonstrating superior performance compared with CT in detecting small tumors and assessing vascular invasion. Li et al. developed and validated an automated MRI-based model for preoperative differentiation between pancreatic squamous cell carcinoma and pancreatic ductal adenocarcinoma using conventional MRI and radiomic features, integrating clinical, radiomic, and hybrid models. The hybrid model combines MRI and radiomic features to distinguish pancreatic squamous cell carcinoma from pancreatic ductal adenocarcinoma (20). Additionally, deep learning-enhanced MR images, assisted by generative adversarial networks (GANs), have shown strong potential in discriminating pancreatic cancer from benign pancreatic diseases (21). Classifiers constructed on the basis of histogram arrays of MR images and CNNs were able to differentiate pancreatic cancer from pancreatic neuroendocrine tumors and solid pseudopapillary tumors, achieving AUCs of 0.896, 0.846, and 0.839 in the training, validation, and test groups (22), respectively. A recent study (23) further demonstrated that the integration of AI, hyperpolarized metabolic magnetic resonance (HP-MR), and multimodality imaging information may facilitate the development of real-time biomarkers for the early detection of pancreatic cancer, assessment of cancer aggressiveness, and early efficacy evaluation. However, HP-MR experiments are currently limited to preclinical models and have not yet been routinely applied in clinical settings.

In summary, with the continuous advancement of artificial intelligence, an increasing number of studies have focused on diagnosing pancreatic cancer using AI-assisted images from various modalities, and some models have demonstrated promising diagnostic efficacy. However, current research remains limited to constructing diagnostic models based on single-modality images combined with artificial intelligence. Different imaging modalities possess distinct advantages and limitations. For example, the sensitivity and specificity of EUS are highly operator dep**e**ndent. Less experienced endoscopists may fail to distinguish subtle imaging differences between early-stage pancreatic cancer and other pathologies (24–26). CT suffers from insufficient soft tissue resolution, radiation exposure, and contrast-related risks, whereas MRI is associated with long examination times, high costs, and numerous contraindications. Given the complexity of pancreatic cancer diagnosis and the inherent limitations of individual imaging modalities, clinicians must comprehensively evaluate multidimensional patient information, including pathological characteristics, baseline physical conditions, and economic affordability. They should carefully weigh the strengths and weaknesses of EUS, CT, MRI, and other imaging techniques to develop personalized and precise diagnostic plans, striving to obtain the most valuable diagnostic information with minimal medical risk. Multimodal medical images harbor rich information beyond mere morphological observations, reflecting not only lesion heterogeneity but also molecular features and prognosis-related data. In contrast, unimodal images inherently lack comprehensive information, limiting the amount of hidden image features that AI can extract. The synergistic integration of EUS, MRI, and CT provides a multidimensional approach to pancreatic cancer detection. EUS demonstrates exceptional sensitivity in identifying subcentimeter lesions, particularly for early-stage tumors undetectable by conventional imaging. Similarly, MRI offers superior soft-tissue contrast resolution, exemplified by sequences such as diffusion-weighted imaging (DWI) and MR cholangiopancreatography (MRCP), enabling precise characterization of parenchymal abnormalities and ductal involvement. Moreover, CT remains indispensable because of its rapid image acquisition, widespread availability, and superior performance in assessing local invasion and distant metastases. This trimodal strategy leverages the unique advantages of each technique, achieving diagnostic accuracy unattainable with any single modality alone. To address this, we propose an innovative strategy to integrate AI with multimodal images (EUS, CT, and MRI). This approach enables systematic mining of quantitative image features of pancreatic cancer across different modalities through high-throughput analysis to accurately elucidate the intrinsic connections between multimodal data and disease biological characteristics. On the basis of this integration, the intelligent diagnostic model for early pancreatic cancer detection, which is constructed by leveraging multimodal information, is expected to significantly enhance diagnostic performance and increase clinical application value.

## 3 Our opinion

Therefore, we propose that AI can utilize diverse types of multimodal image data from patients with pancreatic cancer for extensive AI model training. The aim of this approach is to provide a robust solution for the early diagnosis of pancreatic cancer by identifying non-invasive imaging biomarkers for early pancreatic cancer detection, constructing an end-to-end early screening system for pancreatic cancer based on multimodal images, and implementing a comprehensive intelligent visual diagnosis process from raw data input to clinical decision output.

### 3.1 Synergistic integration of multimodal images

The integration of a patient's EUS, CT, and MR images is a complex multimodal medical image processing task that requires a combination of image alignment, normalization, and fusion techniques. The first step is data preparation and preprocessing. This first step includes data format unification, which ensures that all the images are in the DICOM format (the standard format for medical imaging) or are converted to the DICOM format and checking whether the metadata are complete. Then, spatial resolution and orientation alignment, including resampling and orientation standardization, are performed. Finally, denoising and enhancement are performed. The second step is multimodal image alignment, which aligns images of different modalities to the same anatomical space and extracts anatomical landmarks, such as vascular bifurcations, for ultrasound images to correspond with CT/MRI. The third step is image normalization, which includes intensity normalization and spatial standardization. The fourth step is multimodal image fusion, including pixel-level fusion (displaying bones from CT and soft tissues from MRI superimposed on each other), feature-level fusion (extracting features of different modalities, such as calcified foci from CT and tumor boundaries from MRI, and then fusing them), and body data fusion. The final step is the verification of the fusion effect, in which the clinician evaluates whether the fused image meets the diagnostic needs, and the quantitative metrics include structural similarity (SSIM) and peak signal-to-noise ratio (PSNR). The process of integrating multimodal images must adhere strictly to ethical and privacy regulations.

### 3.2 Building an end-to-end diagnostic model for early pancreatic cancer detection using artificial intelligence and multimodal imaging

We propose that the integration of preprocessed and standardized multimodal data be used to construct an end-to-end early pancreatic cancer diagnostic model. The first step is multimodal data feature extraction, including both radiomic features extracted on the basis of manually delineated regions of interest (ROIs) and features automatically learned end-to-end by deep learning models. Radiomic features are extracted from standardized images using tools such as the PyRadiomics library, 3D Slicer, and ITK-SNAP. The types of extracted features include shape features, texture features, and intensity features. Deep learning automated feature mining requires designing separate branching networks for each modality, which are implemented using the feature extraction layer of the pretrained model. The second step is cross-modal dynamic fusion and feature alignment and splicing, where radiomic features and deep learning features are normalized and spliced into multimodal feature vectors. If the dimensionality of different modal features varies greatly, they can be mapped to a unified dimension through the full connectivity layer. The weights of different modal features are dynamically assigned to suppress redundant information. The third step is end-to-end model integration, where the model architecture requires multibranch inputs, and each modality is independently fed into the branch network. Then, the multimodal features are fused through the attention mechanism. The last step is model validation and interpretation, and the evaluation metrics include the main metrics—AUC–ROC, sensitivity, and specificity—as well as the auxiliary metrics—the Dice coefficient (segmentation task) and attention weight visualization. The whole process faces challenges, such as an insufficient amount of multimodal data, conflicting information between modalities, and limited computer resources. We can use migration learning to synthesize the data, constrained feature space alignment by contrast learning, mixed-precision training, distributed data parallelism, etc., to solve these challenges. Through the above steps, an end-to-end multimodal early pancreatic cancer diagnosis model with high robustness can be constructed.

### 3.3 Application of end-to-end modeling for the early diagnosis of pancreatic cancer in clinical settings

We propose that an end-to-end model for early pancreatic cancer diagnosis can be visualized and integrated into a clinical decision-making system for seamless deployment and practical application. For model visualization, heatmaps can be overlaid on CT and MR images to highlight the tumor regions of interest, assisting physicians in quickly locating lesions. Additionally, 3D segmentation of the detected tumor region can provide more detailed anatomical information. A structured report can be automatically generated, summarizing key indicators such as tumor location, size, morphology, probability of malignancy, and risk of adjacent vascular invasion. To increase model interpretability, the imaging features relied upon by the model can be demonstrated and compared with the diagnostic criteria outlined in clinical guidelines. Multimodel comparisons can also be performed to showcase the predictive ability and consistency of each sub-model. For integration with a PACS, intermediate software based on DCMTK or PyDICOM libraries can be developed to receive DICOM images from the PACS, preprocess them, and input them into the model. Customized plug-ins can be developed for PACS vendors to embed the model results directly into the film-reading interface. To ensure data flow and security, anonymization and encryption of data between the PACS system and the model server are essential. Role-based access control can be implemented, restricting model access to authorized radiologists and surgeons while logging all operations. Physicians should also have the ability to correct ROI and review the basis of model decisions. The clinical decision-making system can incorporate a graded warning system, marking malignancy probabilities with color-coded alerts in the PACS interface and providing suggestions to recommend further tests or follow-up intervals.

Finally, clinical validation of the model is necessary. A multicenter trial involving collaboration with several hospitals can be conducted to calculate the model's sensitivity and specificity and compare its performance against independent diagnoses made by physicians. We believe that in the future, it will be feasible to establish a closed-loop workflow for early pancreatic cancer diagnosis, enabling physicians to efficiently leverage AI models within the PACS environment while adhering to medical protocols and regulatory requirements.

## 4 Challenges in early pancreatic cancer diagnosis using artificial intelligence

Currently, AI technology has demonstrated significant potential for application in the early screening, diagnosis, surgical planning, and prognostic assessment of pancreatic cancer. However, its clinical application still faces several challenges and issues that need to be addressed.

### 4.1 Lack of sufficient evidence

Despite the significant achievements of AI technology in the medical field, the output of AI models remains largely uninterpretable and is often regarded as a “black box (27).” While one can directly observe the model's input data and resulting outputs, understanding the internal data processing mechanisms is challenging. The high accuracy of AI algorithms may come at the cost of reduced interpretability, making it difficult to enhance algorithm performance through modifications to the model's internal structure (28). Consequently, the interpretability of AI-assisted pancreatic cancer diagnostic models remains a critical factor limiting their widespread clinical adoption and represents a key challenge for future AI-related research. We contend that the central limitation of contemporary artificial intelligence models, specifically their lack of interpretability, can be substantially alleviated through the integration of an attention mechanism and feature visualization techniques to establish a dual-analysis framework. The attention mechanism generates heatmaps to precisely identify key anatomical regions of diagnostic importance at the pixel level. For example, in CT lung nodule analysis, the attention mechanism quantitatively highlights malignant feature regions that the model prioritizes, such as spiculation and lobulation. Feature visualization techniques decode the hidden-layer responses of deep neural networks, visually elucidating the model's reasoning process from low-level texture features to high-level semantic concepts. In pathological slide classification tasks, for example, this approach reveals the decision-making rationale for identifying nuclear atypia in cancer cells. This not only strengthens clinicians' confidence in AI-driven decisions but also provides explainable evidence that they comply with medical AI regulatory standards.

### 4.2 Limited training samples

Most pancreatic cancer studies are single-center studies and involve small sample sizes, making them susceptible to selection bias and recall bias. When applied to other centers, these models often result in measurement errors and overfitting, leading to significant fluctuations in accuracy and a lack of stability (29). To address these challenges, it is essential to establish a multi-institutional collaborative framework and conduct prospective, double-blind, multicenter studies. This approach ensures that the training dataset is more representative, thereby enhancing the generalizability and performance of AI models. Additionally, various data augmentation algorithms can be employed to mitigate these issues and effectively increase the diversity and volume of raw data.

### 4.3 Potential selection bias

Despite advancements in the application of artificial intelligence models for pancreatic cancer diagnosis, numerous challenges remain in their clinical implementation. These include a predominance of retrospective studies, issues of confounders and bias, and diagnostic false positives and false-negatives. Additionally, the lack of standardized criteria for evaluating diagnostic accuracy poses a significant obstacle (30, 31). Furthermore, computer-assisted diagnostic systems developed by different researchers across various studies introduce a high risk of selection bias.

### 4.4 Ethical and legal concerns

As an emerging technology, the ethical issues associated with AI must not be overlooked. The use of data should strictly adhere to the principle of informed consent, involving both doctors and patients, which to some extent constrains the application scope of AI technology. In many pancreatic cancer studies, data are anonymized, and the informed consent process is bypassed. However, AI is an evolving and iterative system that requires continuous incorporation of data from new clinical patients. These new patients effectively expose their data to the AI system when it is used for diagnosis and treatment. Finally, there is ongoing debate regarding accountability when AI makes diagnostic errors, which in turn challenges AI-related legislation, regulation, and clinical practice (32). Delineating the responsibilities for AI model development suppliers is urgently needed in such incidents. To address this gap in the discussion and promote the practical application of AI within legal boundaries, introducing and specifically referencing relevant provisions from existing ethical and regulatory frameworks for medical AI is strongly recommended. For example, a thorough examination of the Health Insurance Portability and Accountability Act (HIPAA) regarding liability attribution when AI errors lead to patient data breaches or an analysis of how General Data Protection Regulation (GDPR) provisions concerning automated decision-making, the right to explanation, and liable parties apply to scenarios of medical AI misdiagnosis are needed. By invoking specific clauses from these established regulations, a solid legal foundation and a more comprehensive perspective can be provided for defining the boundaries of developer liability and designing clear dispute resolution pathways.

## 5 Conclusion

In conclusion, as research on AI technology has progressed, there has been a groundbreaking opportunity to transform the diagnosis and treatment paradigms for early-stage pancreatic cancer. For future studies, we propose that AI can be integrated with multimodal medical imaging technologies to develop an end-to-end automated early pancreatic cancer screening system. This would facilitate interoperability between the radiology department's PACS system and the screening system, enabling full-process intelligent visualization diagnostics—from raw data input to clinical decision-making output. Consequently, this approach would allow pancreatic cancer to be detected and treated at its earliest stages, providing a promising pathway to reduce its high mortality rate.

## References

[1] Huang B Huang H Zhang S Zhang D Shi Q Liu J. Artificial intelligence in pancreatic cancer. Theranostics. (2022) 12:6931–54. 10.7150/thno.77949 36276650 PMC9576619

[2] Siegel RL Giaquinto AN Jemal A. Cancer statistics, 2024. CA Cancer J Clin. (2024) 74:12–49. 10.3322/caac.21820 38230766

[3] Erdem S Bolli M Müller SA von Flüe M White R Worni M. Role of lymphadenectomy in resectable pancreatic cancer. Langenbecks Arch Surg. (2020) 405:889–902. 10.1007/s00423-020-01980-2 32902706

[4] Faur AC Lazar DC Ghenciu LA. Artificial intelligence as a noninvasive tool for pancreatic cancer prediction and diagnosis. World J Gastroenterol. (2023) 29:1811–23. 10.3748/wjg.v29.i12.1811 37032728 PMC10080704

[5] Fu Y Lei Y Wang T Curran WJ Liu T Yang X. Deep learning in medical image registration: a review. Phys Med Biol. (2020) 65:20TR01. 10.1088/1361-6560/ab843e 32217829 PMC7759388

[6] Lambin P Rios-Velazquez E Leijenaar R Carvalho S van Stiphout RGPM Granton P. Radiomics: extracting more information from medical images using advanced feature analysis. Eur J Cancer Oxf Engl 1990. (2012) 48:441–6. 10.1016/j.ejca.2011.11.036 22257792 PMC4533986

[7] Pongprasobchai S Pannala R Smyrk TC Bamlet W Pitchumoni S Ougolkov A. Long-term survival and prognostic indicators in small (< or=2 cm) pancreatic cancer. Pancreatology. (2008) 8:587–92. 10.1159/000161009 18849640 PMC2790136

[8] Kocak B Chepelev LL Chu LC Cuocolo R Kelly BS Seeböck P. Assessment of radiomics research (ARISE): a brief guide for authors, reviewers, and readers from the scientific editorial board of European radiology. Eur Radiol. (2023) 33:7556–60. 10.1007/s00330-023-09768-w 37358612

[9] Sakamoto H Kitano M Suetomi Y Maekawa K Takeyama Y Kudo M. Utility of contrast-enhanced endoscopic ultrasonography for diagnosis of small pancreatic carcinomas. Ultrasound Med Biol. (2008) 34:525–32. 10.1016/j.ultrasmedbio.2007.09.018 18045768

[10] Xu M Sethi A. Diagnosing biliary malignancy. Gastrointest Endosc Clin N Am. (2015) 25:677–90. 10.1016/j.giec.2015.06.011 26431597

[11] Kuwahara T Hara K Mizuno N Okuno N Matsumoto S Obata M. Usefulness of deep learning analysis for the diagnosis of malignancy in intraductal papillary mucinous neoplasms of the pancreas. Clin Transl Gastroenterol. (2019) 10:1–8. 10.14309/ctg.0000000000000045 31117111 PMC6602761

[12] Zhang M-M Yang H Jin Z-D Yu J-G Cai Z-Y Li Z-S. Differential diagnosis of pancreatic cancer from normal tissue with digital imaging processing and pattern recognition based on a support vector machine of EUS images. Gastrointest Endosc. (2010) 72:978–85. 10.1016/j.gie.2010.06.042 20855062

[13] Goyal H Sherazi SAA Gupta S Perisetti A Achebe I Ali A. Application of artificial intelligence in diagnosis of pancreatic malignancies by endoscopic ultrasound: a systemic review. Ther Adv Gastroenterol. (2022) 15:17562848221093873. 10.1177/17562848221093873 35509425 PMC9058356

[14] Kuwahara T Hara K Mizuno N Haba S Okuno N Kuraishi Y. Artificial intelligence using deep learning analysis of endoscopic ultrasonography images for the differential diagnosis of pancreatic masses. Endoscopy. (2022) 55:140–9. 10.1055/a-1873-7920 35688454

[15] Cao K Xia Y Yao J Han X Lambert L Zhang T. Large-scale pancreatic cancer detection via non-contrast CT and deep learning. Nat Med. (2023) 29:3033–43. 10.1038/s41591-023-02640-w 37985692 PMC10719100

[16] Mukherjee S Patra A Khasawneh H Korfiatis P Rajamohan N Suman G. Radiomics-based machine-learning models can detect pancreatic cancer on prediagnostic computed tomography scans at a substantial lead time before clinical diagnosis. Gastroenterology. (2022) 163:1435–46.e3. 10.1053/j.gastro.2022.06.066 35788343 PMC12285712

[17] Si K Xue Y Yu X Zhu X Li Q Gong W. Fully end-to-end deep-learning-based diagnosis of pancreatic tumors. Theranostics. (2021) 11:1982–90. 10.7150/thno.52508 33408793 PMC7778580

[18] Ma H Liu Z-X Zhang J-J Wu F-T Xu C-F Shen Z. Construction of a convolutional neural network classifier developed by computed tomography images for pancreatic cancer diagnosis. World J Gastroenterol. (2020) 26:5156–68. 10.3748/wjg.v26.i34.5156 32982116 PMC7495037

[19] Qu C Zeng P Wang H Yuan C Yuan H Xiu D. Application of magnetic resonance imaging in neoadjuvant treatment of pancreatic ductal adenocarcinoma. J Magn Reson Imaging. (2022) 55:1625–32. 10.1002/jmri.28096 35132729

[20] Li Q Zhou Z Chen Y Yu J Zhang H Meng Y. Fully automated magnetic resonance imaging-based radiomics analysis for differentiating pancreatic adenosquamous carcinoma from pancreatic ductal adenocarcinoma. Abdom Radiol. (2023) 48:2074–84. 10.1007/s00261-023-03801-8 36964775

[21] Gao X Wang X. Performance of deep learning for differentiating pancreatic diseases on contrast-enhanced magnetic resonance imaging: a preliminary study. Diagn Interv Imaging. (2020) 101:91–100. 10.1016/j.diii.2019.07.002 31375430

[22] Shi Y-J Zhu H-T Li X-T Zhang X-Y Liu Y-L Wei Y-Y. Histogram array and convolutional neural network of DWI for differentiating pancreatic ductal adenocarcinomas from solid pseudopapillary neoplasms and neuroendocrine neoplasms. Clin Imaging. (2023) 96:15–22. 10.1016/j.clinimag.2023.01.008 36736182

[23] Enriquez JS Chu Y Pudakalakatti S Hsieh KL Salmon D Dutta P. Hyperpolarized magnetic resonance and artificial intelligence: frontiers of imaging in pancreatic cancer. JMIR Med Inform. (2021) 9:e26601. 10.2196/26601 34137725 PMC8277399

[24] Ang TL Kwek ABE Wang LM. Diagnostic endoscopic ultrasound: technique, current status and future directions. Gut Liver. (2018) 12:483–96. 10.5009/gnl17348 29291601 PMC6143442

[25] Harmsen F-JR Domagk D Dietrich CF Hocke M. Discriminating chronic pancreatitis from pancreatic cancer: contrast-enhanced EUS and multidetector computed tomography in direct comparison. Endosc Ultrasound. (2018) 7:395–403. 10.4103/eus.eus_24_18 30246709 PMC6289014

[26] Shahidi N Ou G Lam E Enns R Telford J. When trainees reach competency in performing endoscopic ultrasound: a systematic review. Endosc Int Open. (2017) 5:E239–43. 10.1055/s-0043-100507 28367496 PMC5370237

[27] Katta MR Kalluru PKR Bavishi DA Hameed M Valisekka SS. Artificial intelligence in pancreatic cancer: diagnosis, limitations, and the future prospects—a narrative review. J Cancer Res Clin Oncol. (2023) 149:6743–51. 10.1007/s00432-023-04625-1 36739356 PMC11798185

[28] Lee W Park HJ Lee H-J Jun E Song KB Hwang DW. Preoperative data-based deep learning model for predicting postoperative survival in pancreatic cancer patients. Int J Surg. (2022) 105:106851. 10.1016/j.ijsu.2022.106851 36049618

[29] Hassan C Badalamenti M Maselli R Correale L Iannone A Radaelli F. Computer-aided detection-assisted colonoscopy: classification and relevance of false positives. Gastrointest Endosc. (2020) 92:900–4.e4. 10.1016/j.gie.2020.06.021 32561410

[30] Winkler JK Fink C Toberer F Enk A Deinlein T Hofmann-Wellenhof R. Association between surgical skin markings in dermoscopic images and diagnostic performance of a deep learning convolutional neural network for melanoma recognition. JAMA Dermatol. (2019) 155:1135–41. 10.1001/jamadermatol.2019.1735 31411641 PMC6694463

[31] Esteva A Kuprel B Novoa RA Ko J Swetter SM Blau HM. Dermatologist-level classification of skin cancer with deep neural networks. Nature. (2017) 542:115–8. 10.1038/nature21056 28117445 PMC8382232

[32] Carter SM Rogers W Win KT Frazer H Richards B Houssami N. The ethical, legal and social implications of using artificial intelligence systems in breast cancer care. Breast Edinb Scotl. (2020) 49:25–32. 10.1016/j.breast.2019.10.001 31677530 PMC7375671

